# Replacement Therapy with Blood Products in People Living with HIV

**DOI:** 10.3390/tropicalmed9090213

**Published:** 2024-09-13

**Authors:** Mihaela Cristina Olariu, Mihaela Adela Iancu, Mihai Hristu Olariu, Victoria Aramă, Mădălina Simoiu, Miruna Maria Cruceru, Ecaterina Constanta Barbu, Paul Balanescu, Mihai Lazar

**Affiliations:** 1 Department of Infectious Diseases, “Carol Davila” University of Medicine and Pharmacy, 050474 Bucharest, Romania; mihaela.olariu@umfcd.ro (M.C.O.); victoria.arama@umfcd.ro (V.A.); madalina.simoiu@umfcd.ro (M.S.); 2 “Matei Bals” National Institute of Infectious Diseases, 021105 Bucharest, Romania; ol_mihai@yahoo.com (M.H.O.); miruna.cruceru@yahoo.com (M.M.C.); mihai.lazar@umfcd.ro (M.L.); 3 Department of Internal, Family and Occupational Medicine, “Carol Davila” University of Medicine and Pharmacy, 050474 Bucharest, Romania; plbalanescu@gmail.com; 4 Department of Pathophysiology, Faculty of Medicine, “Carol Davila” University of Medicine and Pharmacy, 050474 Bucharest, Romania; ecaterina.barbu@umfcd.ro; 5 Romania Clinical Research Unit RECIF (Reseau d’Epidemiologie Clinique International Francophone), 020125 Bucharest, Romania

**Keywords:** HIV infection, anemia, thrombocytopenia, CD4+T lymphocyte count

## Abstract

Cytopenias or coagulation deficiencies can occur in people living with HIV (PLWH). The severity of these disorders is influenced by the low levels of CD4+ lymphocytes, viral load, and the stage of viral infection. The aim of our retrospective observational study was to determine the frequency of cytopenias and coagulation deficiencies in PLWH as well as the need for replacement therapy with blood products. We sought to determine whether there is an association between severe anemia or thrombocytopenia (requiring replacement therapy) and CD4+T lymphocyte levels. All 29 patients were critically ill, with 27 out of 29 (93%) in advanced stages of HIV disease and 23 out of 29 (79%) having CD4+ lymphocyte counts below 200 cells/microL. Most patients were either late presenters (45%) or had been lost to follow-up (41%). In addition to HIV infection, various conditions that could alter hematologic parameters were associated, including co-infections with hepatitis viruses, tuberculosis at various sites, malignant diseases, sepsis, SARS-CoV-2 infection, or other opportunistic infections. No significant correlation was found between severe anemia or severe thrombocytopenia or coagulation deficiencies and the CD4+T lymphocyte count. Our data suggest that these hematological disorders in patients with advanced HIV infection are more likely to be associated comorbidities rather than the HIV infection per se.

## 1. Introduction

Cytopenias of different hematopoietic lineages and varying degrees can occur among people living with HIV (PLWH), both in treatment-naive individuals and following the initiation of antiretroviral therapy. The results of a study conducted in South Korea indicated that, among 472 acquiring HIV patients, antiretroviral therapy (ART)-naive patients, lymphopenia was observed in 25.7%, neutropenia in 10%, anemia in 3%, and thrombocytopenia in 2.4%. Additionally, the severity of cytopenia was significantly influenced by the stage of the disease [1].

The severity of anemia, leukopenia/neutropenia, and thrombocytopenia is influenced by the low levels of CD4+ lymphocytes, viral load, and the stage of viral infection [2]. Other authors, in a retrospective analysis, demonstrate that the presence of anemia is associated with decreased survival and disease progression in PLWH, though no direct causal relationship is documented [3]. In light of the discordant data present in the literature, our study aims to provide additional insights to further elucidate these aspects.

Human Immunodeficiency Virus (HIV) can affect the hematopoietic marrow by impacting hematopoietic precursors, resulting in cytopenias of various degrees and across different lineages. In a study conducted on 25 patients who underwent bone marrow biopsy, it was found that, although 21 patients had CD4+ lymphocyte levels below 200 cells/µL and exhibited various cytopenias, 10% of the 25 patients did not show bone marrow involvement [4]. The occurrence of leukopenia, anemia, and thrombocytopenia in these patients can be attributed to various causes: autoimmune etiology, liver diseases with associated hypersplenism, malignant conditions, infections associated with HIV infection, and treatments for diseases associated with HIV infection. Less commonly, changes in hematologic parameters arise as a result of antiretroviral therapies [5].

Drug users, particularly those who consume heroin or cocaine derivatives, may present with coagulation disorders (heroin) or leukopenia (cocaine derivatives). These hematologic abnormalities are attributed to various substances mixed with these drugs to enhance their potency: Brodifacoum, a rodenticide and anticoagulant, in the case of heroin, and Levamisole, a veterinary anthelmintic, in the case of cocaine. The latter has been found in over 65% of analyzed cocaine samples worldwide [6].

Deficiencies in iron, copper, vitamin B12, or folic acid are additional etiologies that should be explored in these patients (Table 1).

The objective of this study was to determine the frequency of cytopenias and other hematologic disorders in acquiring HIV patients and the necessity for replacement therapy with labile blood components in these patients. We aimed to investigate whether anemia and thrombocytopenia requiring replacement therapy are influenced by the CD4+T lymphocyte counts and to identify the associated risk factors.

## 2. Materials and Methods

### 2.1. Patients and Study Design

In our retrospective observational study, we analyzed a total of 607 PLWH hospitalized at the National Institute of Infectious Diseases “Prof. Dr. Matei Balș” over the course of one year (1 January–31 December 2023). Informed consent and General Data Protection Regulation (GDPR) information form were signed by all patients at hospital admittance. Participation in this project did not affect the clinical care and all individual data were kept confidential. Clinical and biological data were gathered using specific Case Report Forms (CRFs), then input into Microsoft Excel 2010 to create a comprehensive study database. Participant information and data were pseudonymized and kept in a secure, offline data-server. The institution holds the decoding list at a separate secure location. All data management practices adhere to the stipulations of Romanian Law 190/2018 on data protection and the European Union General Data Protection Regulation (EU-GDPR) 2016/679. This study was performed in line with the principles of the Declaration of Helsinki and approved by the Ethical Committee of the National Institute for Infectious Diseases “Prof. Dr. Matei Bals” (C06250/18.06.2024).

From this cohort, we selected all consecutive patients who exhibited various cytopenias or hematologic disorders that required replacement therapy with labile blood components. The blood products used included leukoreduced red blood cells (RBCs), whole blood-derived platelets or single donor platelets, fresh frozen plasma (FFP), and cryoprecipitate.

The parameters evaluated included age, sex, years since diagnosis until hospitalization in 2023, number of hospitalization days, disease stage, CD4+T lymphocyte count, viral load, hemoglobin level, leukocyte count, platelet count, prothrombin activity (PA) level, drug use, and patient outcomes (discharge or death). Additionally, conditions associated with HIV infection that may contribute to decreased hematologic parameters requiring correction were also recorded.

We sought to determine whether there is an association between severe anemia or thrombocytopenia (requiring replacement therapy) and CD4+T lymphocyte levels. To demonstrate the relationship between CD4+ lymphocyte count and other clinical and biological parameters, we utilized contingency tables.

We calculated the HIV patients sample considering a confidence level of 95%, a margin of error of 5%, and a population proportion of 50%. Applying the following formula: *n* = [z^2^ × p̂ × (1 − p̂)]/ɛ^2^] (*n* = sample size, z = z score, p̂ = population proportion, ɛ = margin of error), we obtained a minimum sample of 385 patients. We conducted our research on a sample size of 607 patients, finding cytopenia in 29 cases; therefore, we consider our findings relevant for the HIV population.

### 2.2. Definitions

The minimum normal number of CD4+T lymphocytes is 500/microL, and the risk of developing cytopenias of various lineages and degrees increases as immunity declines, specifically with the decrease in CD4+T helper lymphocytes [1,2]. According to the World Health Organization’s (WHO) immunologic classification, CD4+T lymphocyte levels were categorized into three grades: normal count above 500/microL (grade 1), 200–500/microL (grade 2) with mild or moderate HIV—associated immunodeficiency, and below 200/microL (grade 3) with severe immunodepression related to HIV infection [7].

Anemia was defined as a value of hemoglobin less than 13 g/dL in men and less than 12 g/dL in women [8]. In critically ill patients (as was the case for the majority of our patients), anemia requiring therapy was defined as hemoglobin level below 7 g/dL [9].

Thrombocytopenia was defined as a platelet count of less than 150,000/microL [10] and severe thrombocytopenia (<50,000/microL) may require replacement therapy, depending on the underlying disease [11]. The risk of spontaneous bleeding increases in patients with counts below 10,000/microL, while a platelet count < 5000/microL is a hematological emergency [12].

According to current WHO classification and the US Center for Disease Control (CDC), advanced HIV disease includes patients with a CD4+T lymphocyte level below 200/microL or those who have an AIDS-defining event regardless of CD4+ count. Late presenter is defined as the patient with CD4+T cells level < 350/microL at the time of diagnosis or the patient with an AIDS-defining event regardless of CD4+T lymphocyte count [13].

### 2.3. Statistical Analysis

In the descriptive statistics, we employed measures of central tendency (arithmetic mean, mode, median) and measures characterizing the dispersion of data around a central value (range, standard deviation, standard error, percentiles, and coefficient of variation). Normal distribution for continuous variables was evaluated using normality tests (Shapiro–Wilk test). If distribution was not normal, the variables were presented as median and range; otherwise, as mean and standard deviation. Nominal variables were summarized as ratio (percentages). Differences between frequency of categorical variables were evaluated using chi-square tests with computation of odds ratio and 95% confidence interval using online software tools (Medcalc: https://www.medcalc.org/, accessed on 23 July 2024). Difference between continuous variables was evaluated using Mann–Whitney U tests. Correlation analysis was performed by computing Kendall’s Tau coefficient of correlation. Statistical analysis was performed using IBM SPSS Statistics 28 software for Mac OS (IBM Corp., Armonk, NY, USA).

## 3. Results

The analysis included a total of 29 patients, representing 4.8% of the 607 PLWH who required hospital admission in 2023. The characteristics of this cohort are detailed in Table 2.

The median age was 36 years, ranging from a minimum of 24 years to a maximum of 74 years. The distribution of patients by sex showed a predominance of males, with 20 out of 29 (69%). The majority of cases, 23 out of 29 (79%), in both men and women, presented with CD4+T lymphocyte counts below 200/microL at the time of admission.

The measured CD4+ cell counts ranged from 1 cell/microL to 820 cells/microL. Only three patients had normal CD4+T lymphocyte levels, while the remainder had values below the lower limit of normal. The majority of patients (79%) had fewer than 200 CD4+T lymphocytes/microL (Table 3).

The number of blood products used in these patients was as follows: 54 units of leukoreduced red blood cells, 102 units of fresh frozen plasma, 70 units of whole blood-derived platelets or single donor platelets, and 17 units of cryoprecipitate. A total of 19 patients (65%) received leukoreduced red blood cells, 10 patients (34%) received platelets, five patients (17%) received fresh frozen plasma, and four patients (14%) received cryoprecipitate.

Patients presented with a median hemoglobin value of 7.3 g/dL (5.10–14). Of the 29 patients, 28 presented with varying degrees of anemia, though only 19 cases (65.5%) required replacement therapy. In patients transfused with leukoreduced red blood cells, the hemoglobin levels ranged from 5.1 g/dL to 7.8 g/dL with a median value of 6.9 g/dL. We subsequently attempted to determine whether there was an association between low hemoglobin levels (indicative of anemia in patients requiring transfusion) and low CD4+T lymphocyte count in the patients from our case series. A positive correlation, albeit weak, was observed between CD4 cell count and hemoglobin levels (r = 0.30, *p* = 0.02). However, the results indicate that this association was not statistically significant—OR = 2.28, (95% CI: 0.36–14.25) *p* = 0.37 (Table 4).

A total of 18 out of the 29 patients presented with varying degrees of thrombocytopenia and only 10 cases required platelets transfusion. These 10 patients who required replacement therapy with whole blood-derived platelets or single donor platelets had a median platelet count of 16,000/microL, with a minimum of 1000/microL and a maximum of 27,000/microL. There was also no statistically significant association between the degree of thrombocytopenia and CD4+ count in the patients from our case series—OR = 1.06 (95% CI: 0.15–7.14), *p* = 0.94 (Table 4).

No other relevant associations were observed between low CD4 cell count and clinical and biochemical/hematological characteristics.

Fresh frozen plasma (FFP) and/or cryoprecipitate were administered in 7 out of the 29 patients analyzed. In four cases, the deficiency in coagulation factors (measured by prolonged prothrombin time) was attributed to impaired synthetic liver function due to acute viral hepatitis or SARS-CoV-2 infection with fulminant acute liver failure, or acute-on-chronic liver failure in cirrhotic patients with hepatitis virus co-infections associated with HIV infection. Replacement therapy with blood products (FFP, cryoprecipitate) was administered in three cases of disseminated intravascular coagulation (DIC), which occurred in the context of sepsis, most commonly originating from the respiratory tract and, less frequently, from the urinary or digestive tracts. No statistically significant association between the coagulation deficiencies that required replacement therapy and CD4+T lymphocyte count was found in our case series—OR = 0.55 (95% CI: 0.07–3.96), *p* = 0.55 (Table 4).

Among the 29 acquiring HIV patients analyzed, plasma exchange was performed in two cases: one patient with fulminant acute liver failure caused by HBV and another with COVID-19 who developed acute respiratory distress syndrome (ARDS) and subsequently liver failure as part of multiple organ dysfunction syndrome (MODS). Unfortunately, the outcome in both cases was fatal.

Out of the total 29 patients, 6 required replacement therapy with multiple types of blood products. Specifically, one patient received fresh frozen plasma (FFP), platelets, and cryoprecipitate; one patient received leukoreduced red blood cells and cryoprecipitate; one patient received platelets and cryoprecipitate; two patients received RBCs and platelets; and one patient received all four types of products. Additionally, in this last case, a plasma exchange procedure was performed. All 29 patients included in the analysis were critically ill and admitted to the intensive care unit. In eight cases (28%), the outcome was fatal. Of the 29 patients, 25 (86%) were in an advanced stage of HIV infection, and 23 out of 29 (79%) had a CD4 count of less than 200/microL. Regarding the status of HIV infection, only four patients were on antiretroviral therapy (ART). The remaining patients were almost equally split: 13 out of 29 (45%) were newly diagnosed patients (late presenters) and 12 out of 29 (42%) were lost to follow-up patients (LTFU), experiencing immunovirological failure due to non-adherence to treatment. Of the 29 patients, 6 were PWUDs (21%).

In addition to HIV infection, the 29 patients had various conditions that could alter hematologic parameters and require replacement therapy including co-infections with hepatitis viruses, tuberculosis in various locations, malignant diseases, sepsis, SARS-CoV-2 infection, and other opportunistic infections.

Co-infections with hepatitis viruses were present in nine patients. These patients, in various stages of HIV infection, also exhibited chronic hepatitis (five patients), acute hepatitis (one patient with acute hepatitis B), acute HAV hepatitis superimposed on a chronic HCV infection (one case), or two cases with acute-on-chronic liver failure in viral hepatitis coinfection (HBV + HDV or HBV+ HDV + HCV). All reported cases of chronic HCV hepatitis represent active infection, defined by the presence of HCV-RNA. The patient with acute hepatitis B presented concomitant sepsis with a gastrointestinal origin due to *Salmonella Typhi* group DO Vi negative. The HBV-DNA level exceeded 500 million copies. During hospitalization, the patient developed fulminant acute liver failure, which necessitated plasma exchange therapy (two sessions). The patient received replacement therapy with 38 units of fresh frozen plasma (FFP), one unit of cryoprecipitate, and one unit of single donor platelets. Despite these interventions, the patient’s condition deteriorated, resulting in death 10 days after admission.

Active tuberculosis with various manifestations was diagnosed in 7 out of the 29 patients in our cohort. Pulmonary localization prevailed, but there were also two cases with meningoencephalitis in one case and medullary tuberculosis in the other. Regarding the patient diagnosed with medullary tuberculosis, bone marrow biopsy revealed numerous non-necrotizing granulomas and extensive areas of macrophages with vacuolated, gray cytoplasm, accounting for 65% of the surface area. Ziehl–Neelsen staining identified numerous acid-fast bacilli at this site. This patient also had sepsis originating from the abdomen and required replacement therapy with three units of leukoreduced red blood cells. Unfortunately, the outcome was fatal.

Malignant diseases associated with HIV infection were present in five patients, manifesting as Kaposi’s sarcoma in three cases, Hodgkin lymphoma in one patient, and plasmablastic lymphoma, stage IV, refractory to chemotherapy and radiotherapy, in one case.

Sepsis with MODS was present in nine patients, with origins including cutaneous (two cases), urinary (one patient), abdominal (two cases), and most frequently, pulmonary sites (four patients). The isolated pathogens included fungi, Gram-negative bacilli, and Gram-positive cocci. Eight patients were in the advanced stage of HIV infection, and the outcome was fatal in seven of the cases with sepsis. Five patients presented with pneumocystis pneumonia, resulting in death in three cases, while two had a favorable outcome. One patient presented with extrapulmonary cryptococcosis, manifesting as cryptococcal meningitis and cutaneous cryptococcosis, and had a favorable outcome under treatment.

One patient presented with a SARS-CoV-2 infection and subsequently developed ARDS during hospitalization, requiring ventilatory support with intubation and mechanical ventilation (IOT-VM) as well as extracorporeal membrane oxygenation (ECMO) and dialysis. Despite treatment, the patient’s condition deteriorated, leading to acute liver failure, for which multiple plasma exchange sessions were performed. During the 26-day hospitalization, this patient received 18 units of leukoreduced red blood cells, 46 units of fresh frozen plasma, 35 units of whole blood-derived or single donor platelets, and two units of cryoprecipitate.

## 4. Discussion

In our cohort, although only a small number (29 out of 607 HIV patients hospitalized over a one-year period in a dedicated care center) presented with severe cytopenias or coagulation deficiencies requiring replacement therapies, all of these cases were critically ill patients needing intensive care.

All 29 patients who received blood products had multiple opportunistic coinfections or comorbidities that required intensive care treatment, and 8 of them had a fatal prognosis. Almost all patients (27 out of 29 or 93%) were in advanced stages of HIV disease, and 23 out of 29 (79%) had CD4+T lymphocyte counts below 200 cells/microL. Most patients were either late presenters (45%) or LTFU (41%). HIV/AIDS late presentation represents a significant burden on health services due to the high rates of morbidity and mortality, as well as the increased risk of transmission [14]. Additionally, these patients are at an elevated risk of developing treatment resistance. These factors collectively lead to substantial healthcare costs [15].

In our study, six patients were people who use drugs (PWUDs), all of whom had advanced HIV infection. The immunosuppressive effect of opioids on immune system cells has been demonstrated in several in vitro studies [16,17], while in vivo studies have reported that, in HIV/AIDS patients, opioid use is associated with higher HIV RNA levels and accelerated disease progression [18,19,20]. The results of a recent 2023 study demonstrated that, in vivo, opioids do not appear to directly influence the replication, latency, or reactivation of HIV in CD4+T lymphocytes. However, in vivo, opioid use has a systemic immunosuppressive effect that contributes to the maintenance of a latent HIV reservoir [21]. Drug use negatively influences adherence to treatment and access to medical care [22].

All patients had complications or comorbidities that could lead to alterations in hematologic parameters, necessitating replacement therapy. The most common hematologic deficiencies requiring replacement therapy were anemia (19/29) and thrombocytopenia (10/29).

There are multiple causes of cytopenias in PLWH. Primarily, HIV has a suppressive effect on the bone marrow [23]. HIV inhibits the proliferation of hematopoietic progenitor cells (HPCs), disrupts erythroid differentiation, and impairs the maturation and differentiation of megakaryocytes. Chronic dysregulation of cytokines induced by HIV infection also exerts a negative impact on hematopoietic progenitor cells (HPCs) [24].

In the advanced stages of the disease, additional causes and pathogenic mechanisms of anemia arise, such as opportunistic infections, bleeding, malnutrition, hypersplenism in advanced liver disease due to viral co-infections, and the side effects of ARV medication [25,26]. At the same time, anemia is a predictor of disease progression in PLWH [27]. Several studies have shown that anemia in HIV patients is closely related to low CD4 +T lymphocyte levels, advanced stage of the disease, opportunistic infections, as well as age and sex [24].

Similar to anemia, thrombocytopenia is a predictor of disease progression. HIV infection causes thrombocytopenia through two primary etiopathogenic mechanisms: the peripheral destruction of platelets through the cross-reaction of antibodies in the early stages of the infection and the decreased platelets production in the advanced stages [28]. The clinical consequences of immune-mediated platelet destruction are idiopathic thrombocytopenic purpura or immune thrombocytopenic purpura [29]. In the bone marrow, HIV disrupts megakaryocytic maturation and induces structural changes in megakaryocytes which subsequently increase apoptosis of these cells [24].

Opportunistic infections, co-infections with hepatitis viruses, and neoplasms associated with HIV infection are additional causes of cytopenia in PLWH. Opportunistic infections and/or neoplasms can infiltrate the hematopoietic marrow, leading to severe cytopenias in PLWH, particularly in those with advanced stages of the infection. In cases wherein bone marrow infiltration is suspected, performing a bone marrow biopsy is mandatory.

In cases of co-infections with hepatitis viruses, liver damage leads to decreased production of thrombopoietin and, due to hypersplenism, increased destruction of platelets [30]. Some studies have also reported the prognostic value of scores based on CBCs as predictive factors for adverse outcomes in different viral infections, including SARS-CoV-2 [31,32].

However, our statistical data did not reveal a significant correlation between the level of these cytopenias and the CD4+T lymphocyte count. A plausible explanation for this finding could be the small sample size (19 out of 29 patients with anemia and only 10 out of 29 patients with thrombocytopenia required substitution treatment); a larger cohort of patients would be required to achieve a statistically significant result. Our data suggest that these cytopenias in patients with advanced HIV infection are attributable to associated comorbidities rather than the HIV infection per se. Thus, opportunistic infections (tuberculosis, pneumocystis), neoplasms, and co-infections with hepatitis viruses leading to advanced liver disease are the primary causes of severe anemia or thrombocytopenia in these patients.

People living with HIV (PLWH) exhibit a prothrombotic status primarily driven by chronic inflammation, endothelial cell dysfunction, platelet activation, and coagulation factor activation [33,34]. Platelet activation can also be triggered by opportunistic coinfections or neoplasms associated with HIV infection [35]. In our study, no thrombotic events were documented. Fresh frozen plasma and cryoprecipitate were administered in nine patients who required supplementation of coagulation factors (due to prolonged prothrombin time), a deficiency that arose either from impaired synthesis (acute hepatitis, liver failure) or increased consumption (disseminated intravascular coagulation syndrome). No significant correlation was found between coagulation factors deficiency that required replacement therapy and CD4+T lymphocyte level.

The limitations of this study include the small sample size and the retrospective nature of the analysis. Further research is warranted to prospectively evaluate this category of patients, incorporating a larger number of cases. Additionally, an analysis of the care and hospitalization costs would allow for an improvement in health policies for PLWH in advanced stages of the disease.

## 5. Conclusions

Advanced stages of HIV infection may present with cytopenias (or combinations thereof) and coagulation factor deficiencies that require specific replacement therapies. In most cases, multiple contributing factors are seen, including associated conditions such as opportunistic infections, viral hepatitis co-infections, and neoplasms, rather than a direct causal relationship with the HIV virus or the immunosuppression induced by it (decrease in CD4+T lymphocyte levels).

Most hematological manifestations, like anemia and thrombocytopenia, are treatable conditions. The decision to administer blood products to the PLWH must be individualized, tailored to the clinical status, the biological evaluation, and the recording of all the causes that can generate significant cytopenias or coagulation disorders.

The profile of such a clinical case is that of a critically ill patient with severe immunosuppression and multiple organ failures. Correction of hematologic deficiencies and/or coagulation disorders must be performed promptly with appropriate products and in adequate quantities, in dedicated centers.

## Figures and Tables

**Table 1:** Etiology of cytopenias which can occur among PLWH.

Causes of Cytopenias	Observations
Viral infections:	
HIV, CMV, EBV	Central origin (viruses affecting the hematopoietic marrow) as well as peripheral causes
Parvovirus B19	Can cause severe anemia—aplastic anemia
Hepatitis Viruses: HAV, HBV, HCV	Direct action on the hematopoietic marrow or through hypersplenism in advanced liver disease
Infections with opportunistic pathogens: *Cryptococcus neoformans*, *Mycobacterium avium*, *Mycobacterium tuberculosis*, *Histoplasma capsulatum*	Hematopoietic marrow involvement
Parasitic Infections: *Toxoplasma gondii* (rare)	Bone marrow involvement and/or septic shock
Sepsis	Direct action of pathogens, DIC onset and/or bone marrow involvement
Medications (antibacterial, antiviral, antifungal):	Attention to medications that can cause hemolytic anemia in G-6PD deficient patients
Cephalosporins	Primarily leukopenia
Sulfonamides	Anemia, pancytopenia
Pyrimethamine	Pancytopenia
Vancomycin	Leukopenia
Linezolid	Anemia, thrombocytopenia, less frequently leukopenia
Ganciclovir/Valganciclovir/Foscarnet	Anemia, leukopenia, more frequently thrombocytopenia
Zidovudine	Macrocytic anemia (tetrahydrofolate blockage)
Neoplasms: leukemia, lymphoma/neoplasms with metastasis/Kaposi’s sarcoma/Castleman disease	Cytopenias of various degrees due to bone marrow involvement and/or antineoplastic treatments
Autoimmune Causes	
Autoimmune Hemolytic Anemia/Immune Thrombocytopenia	Anti-erythrocyte/anti-thrombocyte antibodies; Anti-leukocyte antibodies may also be present.
Hemophagocytic lymphohistiocytosis	Rare, severe pancytopenia, fever, neurological impairment, DIC.
Inflammatory State Associated with HIV/Chronic Infections	Chronic anemia (hypochromic, normocytic, or microcytic)
People who use drugs (PWUDs)	
Synthetic Cannabinoids (“K2”, “spice”) contaminated with Brodifacoum	Coagulation disorders, hemorrhagic syndrome
Cocaine contaminated with Levamisole	Leukopenia with neutropenia, hemorrhagic bullae
Mineral and/or Vitamin Deficiencies:	
Iron	Anemia
Copper/Folic Acid/Vitamin B12	Pancytopenia of varying degrees
Microangiopathic hemolytic anemia due to infections or medications	Severe anemia and thrombocytopenia, neurological manifestations, fever, renal insufficiency

HIV—Human Immunodeficiency Virus, HAV—Hepatitis A Virus, HBV—Hepatitis B Virus, HCV—Hepatitis C Virus, CMV—Cytomegalovirus, EBV—Ebstein Barr Virus, DIC—Disseminated Intravascular Coagulation.

**Table 2:** Descriptive data of the cohort *.

Parameter	Total Number of Patients (*n* = 29)
Gender (male), *n* (%)	20 (69%)
Age (years)	36 (24–74)
Years since first diagnosis (years)	3 (0–35)
Days of hospitalization (days)	22 (2–193)
Viral load (copies/mL) (data for 26 patients)	22,350 (0–3,160,000)
Deceased, *n* (%)	8 (27.6%)
CD4 cells (/microL)	44 (1–820)
Low CD4 cells (<200 cells/microL), *n* (%)	23 (79.3%)
Hemoglobin (g/dL)	7.3 (5.10–14)
Anemia, *n* (%)	28 (96.6%)
Anemia requiring therapy, *n* (%)	19 (65.5%)
Leukocyte count (/microL)	3700 (1200–11,000)
Platelet count (/microL)	60,000 (0–374,000)
Thrombocytopenia, *n* (%)	18 (62%)
Thrombocyte replacement therapy, *n* (%)	10 (34.48%)
Infection status	Advanced HIV infection 27/29 (93.1%) Late presenter 13/29 (44.82%) Lost to follow-up (LTFU) 12/29 (41.37%)
Viral hepatitis coinfection, *n* (%)	9 (31%)
Tuberculosis, *n* (%)	7 (24.1%)
Malignancies, *n* (%)	5 (17.2)

* Continuous data are presented as median and range. Categorical data are presented as number and percent.

**Table 3:** Distribution of patients according to CD4+T lymphocytes level.

CD4+T Lymphocytes Count	Female 9 (31%)	Male 20 (69%)	Total Number of Patients 29
Grade 1 (>500/microL)	2	1	3 (10.34%)
Grade 2 (200–500/microL)	1	2	3 (10.34%)
Grade 3 (<200/microL)	6	17	23 (79.31%)

**Table 4:** Association between hematological disorders and CD4+T lymphocyte count in PLWH.

	CD4 < 200/microL *n* = 23	CD4 ≥ 200/microL *n* = 6	Statistical Analysis
Anemia requiring therapy	16 (69.6%)	3 (50%)	OR = 2.28, (95% CI: 0.36–14.25) *p* = 0.37 *
Thrombocytopenia requiring therapy	8 (34.8%)	2 (33.3%)	OR = 1.06 (95% CI: 0.15–7.14), *p* = 0.94 *
Coagulation deficiencies requiring FFP and/or cryoprecipitate	5 (21.7%)	2 (33.3%)	OR = 0.55 (95% CI: 0.07–3.96), *p* = 0.55 *

PLWH—people living with HIV; * Chi-square test.

## Data Availability

The raw data supporting the conclusions of this article will be made available by the authors on request.
